# Buffalo Medical Association

**Published:** 1879-10

**Authors:** 


### Society Reports.

### BUFFALO MEDICAL ASSOCIATION.

*Stated Meeting, September 2, 1879.*

DR. THOS. LOTHROP, PRESIDENT PRO TEM., IN THE CHAIR.

The Association met, with the following members in attendance: Drs. Rochester, Trowbridge, Phelps, Lothrop, Keene, Mynter, Brecht, Bartlett, Hauenstein, Fowler. In the absence from the city of the President, Dr. Lothrop was elected President pro tem.

A ballot was taken on the application of Dr. A. R. Davidson, and he was declared elected a member of the Association.

Dr. Herman Mynter read a paper on Perityphlitic Abscess. (See Original Communication.)

DISCUSSION.

Dr. Hauenstein said the paper had interested him very much. He had treated quite a number of cases of typhlitic and perityphlitic abscess, and had witnessed several post-mortem examinations. In some of these was found perforation of the appendix, and in others a confused mass of inflammatory products, surrounding a central point of ulceration. He was able to call to mind a child eight or nine years old, where the abscess made a spontaneous opening externally, discharging pus, mixed with fecal matter. The parts healed and the child recovered. In another case of a similar character the abscess opened below Poupart's ligament, death ensuing.

Sometimes it is very difficult to diagnose the true character of the disease. Often the physician is not called in till after the inflammation has become diffused over the whole, or greater part of the abdomen. He remembers being called to see a young lady of about nineteen years of age, on the ninth day of her sickness with symptoms of general peritonitis, and it was almost impossible to determine where the trouble began. An autopsy showed that it had its origin in or about the appendix vermiformis. He was unable to see how an operation could have been of any use in this case.

Professor Rochester, in the course of his remarks, said it gave him much pleasure to be present at this meeting, and hear Dr. Mynter's paper, which he could fully endorse. It called up a subject in which he had taken very much interest. In his professional experience he had met with twenty-three cases of perityphlitic abscess, seventeen of which were fatal.

The surgical treatment of perityphlitic abscess was not novel. Dr. Willard Parker operated twenty-five years ago or more. Prior to that time it had received similar treatment at the hands of English surgeons, but subsequently fell into disuse. Of late years a great many cases have been reported in the journals. The diagnosis of this form of disease is often made with great difficulty. The appendix of itself possesses very little vitality, and when in a healthy condition excludes the presence of any foreign substance. Rokitansky, whose pathological experience is probably unrivaled, speaks fully of this.

The appendix becomes the seat of a chronic inflammation, which gradually enlarges its orifice, permitting foreign bodies to enter. These may be of various kinds—strawberry seeds, cherry stones, or other small substances; but if the bodies usually present be carefully examined, they will nearly always be found to be gall-stones. The doctor exhibited an appendix, in which was embedded what appeared to be a bean, but examined more closely, proved to be a gall-stone. The claw of an oyster has been noticed in addition to the other foreign substances already mentioned.

He believes there often exists a chronic inflammatory state of the appendix for an indefinite period, which at some future time becomes the depository of a foreign body, around which concretions gather, until an abscess forms. This condition is often associated with pain in the right leg, as in strangulated hernia, the limb being drawn up.

The pain is often severe, but may subside, and no inconvenience follow. Then, in about a year, another attack comes on of a similar character. He had observed a number of such attacks succeed each other in the same individual before a fatal termination. All of his cases, except one, occurred between the ages of ten and twenty years, the exceptional one being fifty years old. There was a slight preponderance of the male sex.

His treatment of the disease was strictly anodyne, avoiding cathartics. He relied upon opium and warm applications, but he remembered one case in which castor oil was freely used, and the patient got well. It was that of a young officer, a graduate of West Point Military Academy, who came under his care while suffering from this form of disease. He thought there could be no mistake in the diagnosis. He had been treated by Dr. G. W. Fisher, of Peekskill, N. Y., who made a like diagnosis. Dr. Fisher's treatment consisted of an half to one ounce of castor oil every second to third night, and opium at bed-time. This treatment was continued, and he fully recovered.

This is the only case he ever knew to get well under the purgative treatment. The cases we usually meet with are frequently preceded by diarrhœa; vomiting usually occurs; pulse about 110, temperature 102 to 104; pain in the right iliac region, perforation and death.

Should we detect a well-defined circumscribed abscess, it would be safe to operate; do not think it wise to plunge a trocar into the swelling. The operation should be regarded a delicate one, as much so as tying the iliac artery. It can be made without much danger.

Dr. Rochester was called to see a case which was thought to be suffering from colic. Disease of the appendix was diagnosed. He was treated with opium, and died on the fifth day.

At the autopsy was found thickening, swelling, and perforation of the appendix; was of the opinion that it had been subject to catarrhal inflammation; he could not understand how a surgical operation would have benefited his patient. Surely we would not think of ligating the appendix.

As a rule the perforation takes place between the foreign body and attachment of the appendix to the cæcum; had one case where it opened at the extreme end, the opening communicating with the bladder and rectum. This might have been a favorable case to operate upon. In acute cases there is doubt of the value of an operation, unless a circumscribed swelling can be detected.

It must be borne in mind that these cases sometimes get well by a spontaneous evacuation. In two cases the symptoms subsided, and the patients were lost sight of. In one case, that of a boy who was kicked by another boy, causing disease of appendix, and death, the appendix was found gangrenous. The specimen was shown the members present. He could endorse Dr. Mynter's paper, and should recommend an operation if a local tumor could be detected.

A few years ago, Dr. Miner operated successfully for perityphlitic abscess. The patient had been ill for many years, and for a long time had complained of pain in the right iliac region. The abscess was circumscribed and could be readily detected.

Dr. Phelps said he made a post mortem examination of two or three of Dr. Rochester's cases. In the appendix of one was found a piece of straw. He was called to see a case last December. The patient, a boy, was wakened at midnight with severe pain in the right iliac region. A tumor was readily detected. Called Dr. Rochester in to see him the following afternoon. He was treated with opium, sat up in about a month, and recovered. Occasionally he comes to see me, complaining of pain in that region, and it is my practice to keep his bowels well open.

Dr. Mynter, in his closing remarks, said that in cases in which the disease commenced with an universal peritonitis, or in which universal peritonitis set in during the first days, no one would think of operating, although he believed the time would come, when we, in such cases, would open the abdominal cavity and ligate the appendix vermiformis. The operation was now recommended in those cases only in which a distinct local tumor was felt, the meaning of the operation was, strictly speaking, to establish an artificial anus, and then leave the rest to nature.

Intermittent fever was reported to be more prevalent in this city than for many years.

The Association then adjourned.

